# Benefits of Rebuilding Global Marine Fisheries Outweigh Costs

**DOI:** 10.1371/journal.pone.0040542

**Published:** 2012-07-13

**Authors:** Ussif Rashid Sumaila, William Cheung, Andrew Dyck, Kamal Gueye, Ling Huang, Vicky Lam, Daniel Pauly, Thara Srinivasan, Wilf Swartz, Reginald Watson, Dirk Zeller

**Affiliations:** 1 Fisheries Economics Research Unit, Fisheries Centre, University of British Columbia, Vancouver, British Columbia, Canada; 2 Sea Around Us Project, Fisheries Centre, University of British Columbia, Vancouver, British Columbia, Canada; 3 The United Nations Environment Programme, Geneva, Switzerland; 4 Department of Economics, University of Connecticut, Storrs, Connecticut, United States of America; 5 Pacific Ecoinformatics and Computational Ecology Lab, Berkeley, California, United States of America; University of Western Australia, Australia

## Abstract

Global marine fisheries are currently underperforming, largely due to overfishing. An analysis of global databases finds that resource rent net of subsidies from rebuilt world fisheries could increase from the current negative US$13 billion to positive US$54 billion per year, resulting in a net gain of US$600 to US$1,400 billion in present value over fifty years after rebuilding. To realize this gain, governments need to implement a rebuilding program at a cost of about US$203 (US$130–US$292) billion in present value. We estimate that it would take just 12 years after rebuilding begins for the benefits to surpass the cost. Even without accounting for the potential boost to recreational fisheries, and ignoring ancillary and non-market values that would likely increase, the potential benefits of rebuilding global fisheries far outweigh the costs.

## Introduction

Fish are among the planet’s most important renewable natural resources. Beyond playing a crucial role in marine ecosystems, fish support human well-being through employment in fishing, processing, and retail services [1]–[3], as well as food security for the poor, particularly in developing countries [4]. Overexploitation [3], [5], [6] and rising ocean temperatures threaten global fisheries [7]–[9]. As demonstrated by the collapse of northern cod off Newfoundland, the depletion of fish stocks can have devastating effects on human well-being [10], [11]. As human populations continue to grow, the future benefits that fishery resources can provide will depend largely on how well they are rebuilt and managed. However, policy makers often perceive that rebuilding fisheries is too expensive in the short-term and therefore avoid taking the necessary actions to sustainably manage fish stocks. Therefore, a crucial question for policy makers is what is the potential net economic benefit of rebuilding global fisheries? Here, we address this question on a global scale.

Fisheries economists use resource rent (i.e., what remains after fishing costs and subsidies are deducted from revenue) as an indicator of fisheries performance [12], although others argue that this is inadequate because it does not capture all the benefits derived from marine fisheries [13]. Here, we adhere to using resource rent as our primary indicator of economic performance, but we also report payments to labor (i.e., wages) and earnings to fishing companies as additional indicators of fisheries benefits. With these additional indicators, we recognize that fishing capacity is not often converted to other uses easily (i.e., it is non-malleable) and that the opportunity cost of fishing labor (i.e., the alternative wages that fishers can earn if they did not fish) in many fishing communities is low due to a dearth of alternative employment. Even with these additional indicators, other important contributions of fish populations to the economy, such as the value created through the production chain [1] and non-market values [14] are not captured.

Over the past decade, we have gathered data on the economics of global fisheries from a range of sources, including scientific, economic, governmental, inter- and non-governmental publications, to create several global databases on catch [15]; ex-vessel fish prices [16]; subsidies [17]; and fishing costs [18] (Tables S1, S2, S3, S4, S5 and S6). From these databases, we compile landed value of catch, cost of fishing, payments to labor, earnings of fishing companies, and fisheries subsidies for 144 maritime countries of the world. We then compute both current and potential maximum resource rent, wages, and earnings to fishing enterprises.

## Results

### 1. Gains from Rebuilding

**Figure 1:**
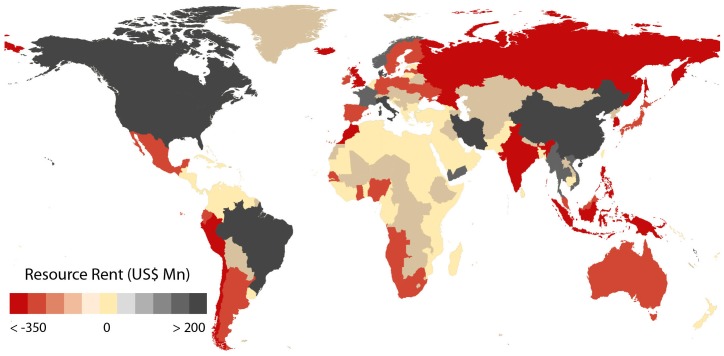
Summary of resource rent (adjusted for subsidies) from current fisheries. We see that several countries are in red once the full cost of fishing, including harmful subsidies are taken into account.

**Figure 2:**
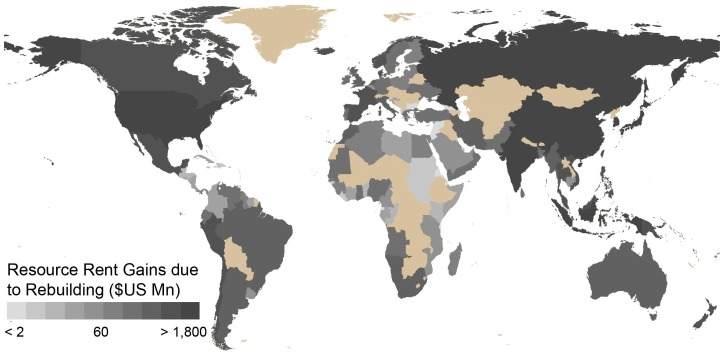
Summary of resource rent (adjusted for subsidies) from rebuilt fisheries (rent in all maritime countries increase after rebuilding).

Global marine fisheries landings are projected to average 89 million t per year (range 83–99 million t) (Table 1) when rebuilt [19], with a corresponding mean landed value of US$101 billion per year (range US$93–116 billion). The wide ranges help to address uncertainties about the magnitude of global overfishing currently debated in the literature (as discussed in Materials and Methods). The cost of fishing in this rebuilt scenario is estimated at US$37 (US$29–44) billion compared to US$73 (US$50–96) billion per year currently. Returns to capital invested (i.e., normal profit) and payments to labor would amount to US$3 (US$2–4) billion and US$16 (US$12–19) billion per annum, respectively, while resource rent from rebuilt global fisheries would be US$54 (US$39–77) billion per year (summary of current resource rent is displayed by country in Figure 1, with details in Table 1). (The *Sunken Billions* report of the World Bank [20], which estimated economic rent without addressing the cost of reform, arrived at a potential resource rent of US$50 billion per year, using a different approach.) Gains in resource rent from the current situation to a rebuilt global fishery would be US$66 (US$51–89) billion a year, while wages and returns to capital will decrease to US$16 and US$3 billion, respectively (Table 1). Figure 2 summarizes the net gains in resource rent by maritime country.

**Table 1:** Key economic figures of global fisheries.

Key indicators, annual data (unit)	Current	Rebuilt fisheries		
		Lower bound	Mean	Upper bound
Catch (t)	80.2	82.7	88.7	99.4
Catch value (US$ billions)	87.7	92.6	100.5	116.3
Variable fishing cost (US$ billions)	73.0	43.9	36.6	29.3
Normal profit (US$ billions)	6.1	3.7	3.0	2.4
Wages (US$ billions)	31.0	18.6	15.5	12.4
Subsidies (US$ billions)	27.2	10.0	10.0	10.0
Rent net of subsidies* (US$ billions)	−12.5	39.0	54.0	77.0
Rent increase over current values (US$ billions)	–	51.2	66.4	89.4
NPV of resource rent increases (US$ billions)	–	665.2	972.0	1,428.1
Transition costs** (US$ billions)	–	129.9	202.9	292.2
NPV net of transition costs (US$ billions)	–	535.3	769.1	1,135.9

NPV: Net Present Value.

* The (resource) rent is the return to ‘owners’ of fish stocks, which is the surplus from gross revenue after total cost of fishing is deducted and subsidies taken into account.

** Transition costs include the costs to society of reducing current fishing effort to levels consistent with maximum sustainable yield and the payments governments may decide to employ to adjust capital and labour to uses outside the fisheries sector. Such payments may include vessel buyback programs and alternative employment training initiatives for fishers.

### 2. Cost of Reform

**Figure 3:**
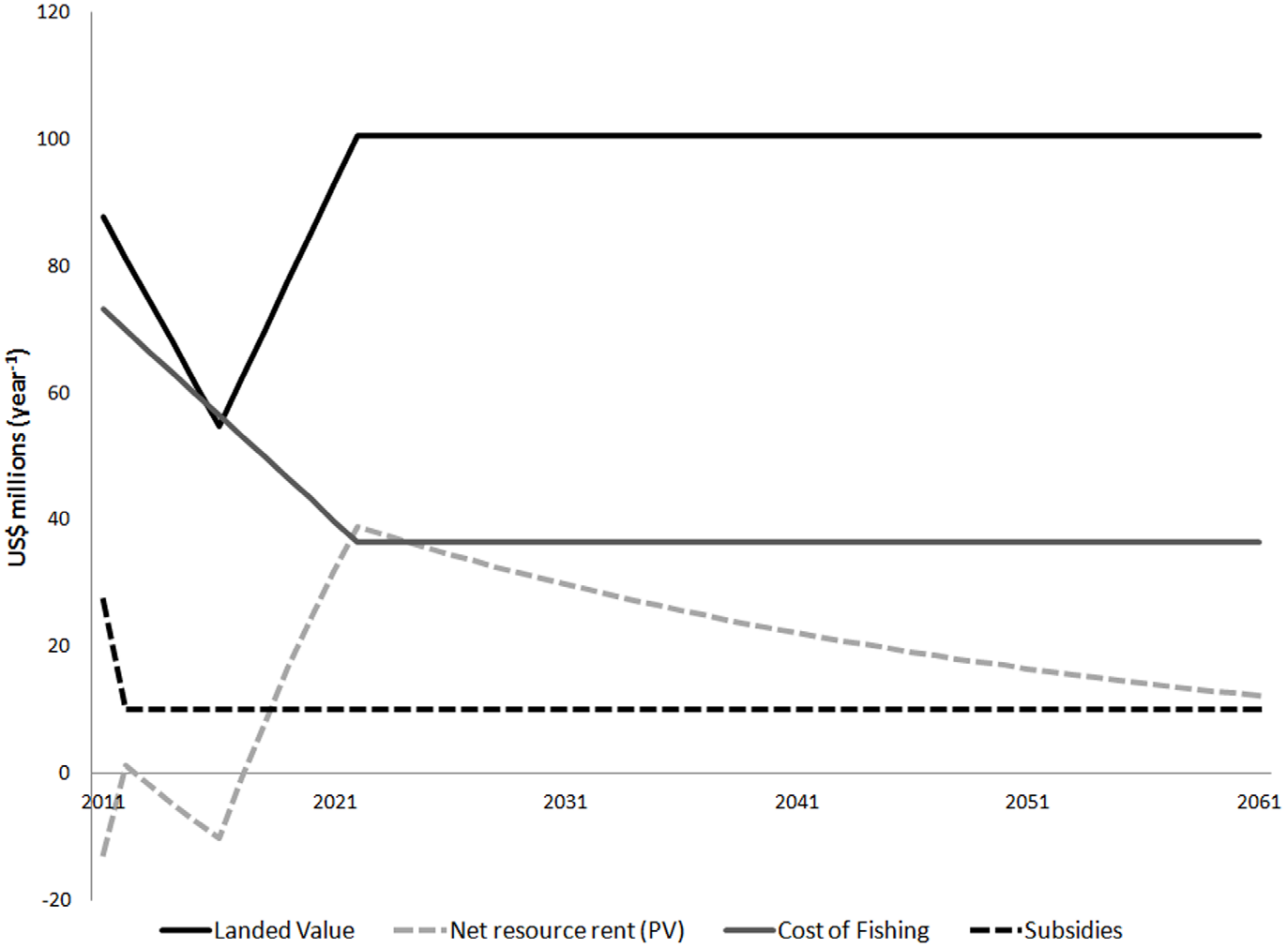
Transition time path of key rebuilding global fisheries variables.

The real cost to society of rebuilding fisheries, once the elimination of an estimated US$19 billion per year of harmful and ambiguous subsidies is taken into account [17], is negative, implying that society as a whole will make money by engaging in rebuilding (Figure 3). However, fishing enterprises and fishers will lose profits and wages during rebuilding. Hence, to implement a rebuilding reform, governments may need to temporarily invest extra resources to mitigate these impacts.

The world’s current fishing capacity is estimated to be up to 2.5 times more than what is needed to land the Maximum Sustainable Yield (MSY) [21]. This suggests that to rebuild global fisheries, we need to trim excess capacity from the current 4.3 million fishing boats [3]. Assuming that current capacity is between 1.5 and 2.5 times the level needed to maximize sustainable catch, fishing effort needs to be reduced by between 40 and 60 per cent, or up to 2.6 million boats. Fisheries currently employ more than 35 million people globally [3]. If we simplify by assuming linearity between boats and people, this implies that between 15 and 22 million fishers would need to be moved to other livelihood activities in order to rebuild global fisheries. This is a challenge, but one that is surmountable. For instance, even though in some fisheries most fishers may see fishing as a way of life and therefore may not want to exit fishing [22], it has been reported that up to 75% of fishers in Hong Kong would be willing to leave the industry if suitable alternatives or compensation were available [23]. Similar sentiments are likely to also occur in many other countries. In any case, it is better to undertake this transition as part of a rebuilding policy rather than having it forced upon us through loss of resources [10], [11].

Using the unit cost of reducing fishing effort calculated in Materials and Methods, the total amount that governments need to invest to rebuild world fisheries ranges between US$130 and US$292 billion in present value, with a mean of US$203 billion. This total transition cost would be spread over the time required to rebuild fisheries within each country.

### 3. Net Gain from Rebuilding

Global fisheries are not living up to their revenue potential; the total cost of fishing is too high and governments provide harmful subsidies to the sector, which results in a negative resource rent (i.e., economic loss to society) of about US$13 billion per year (Table 1). Rebuilding would result in a gain in resource rent of US$66 billion per year, which when discounted over the next 50 years using a 3 per cent real discount rate, generates a present value of between US$660 and US$1,430 billion (Table 1), i.e., between 3 and 7 times the mean cost of fisheries rebuilding reform. Furthermore, it would likely take just 12 years after rebuilding efforts begin for the gains to exceed the costs of adjustment (Figure 3). A higher discount rate will reduce the present value of gain from rebuilding and increase the time needed to balance the gain with the costs of adjustment, and *vice versa* (see Materials and Methods for the justification of a 3% discount rate). Our results suggest that, even without accounting for the potential boost to recreational fisheries, processing, retail and non-market values that would likely increase, there is a substantial net economic benefit to be derived from rebuilding global fisheries, with net gains large enough to compensate for uncertainties in our assumptions and estimates. Rebuilding fisheries makes good business sense. The challenge is how to move global fisheries from their current dismal economic state to a more prosperous one.

## Discussion

Even though the overall results we present are consistent with other estimates about the extent of subsidies, excess fishing pressure and the potential for increased biological yield, the country-by-country analysis (Tables S1, S2, S3, S4, S5 and S6) may reveal results that differ from expectations. This is not surprising, as our analysis produces estimates with ranges, and therefore computing midpoint estimates may over- or underestimate numbers for some countries. This is more likely to happen for small developing countries where observed data are limited, and we therefore had to rely on statistical methods to produce estimates for these countries. The key to improving our estimates is for the collection of economic data for fisheries to be given priority by maritime countries.

There are also situations where large maritime countries (e.g., Peru, Chile, and Indonesia) may show counterintuitive estimates. Similarly unexpected results for countries such as Australia and Iceland, known to have good fisheries management regimes were found. In these cases too, the result of the statistical estimation required in the absence of observed or collected, publicly available country-specific data may be a reason. However, the reported results may indeed be correct yet unanticipated, as explained below.

**Figure 4:**
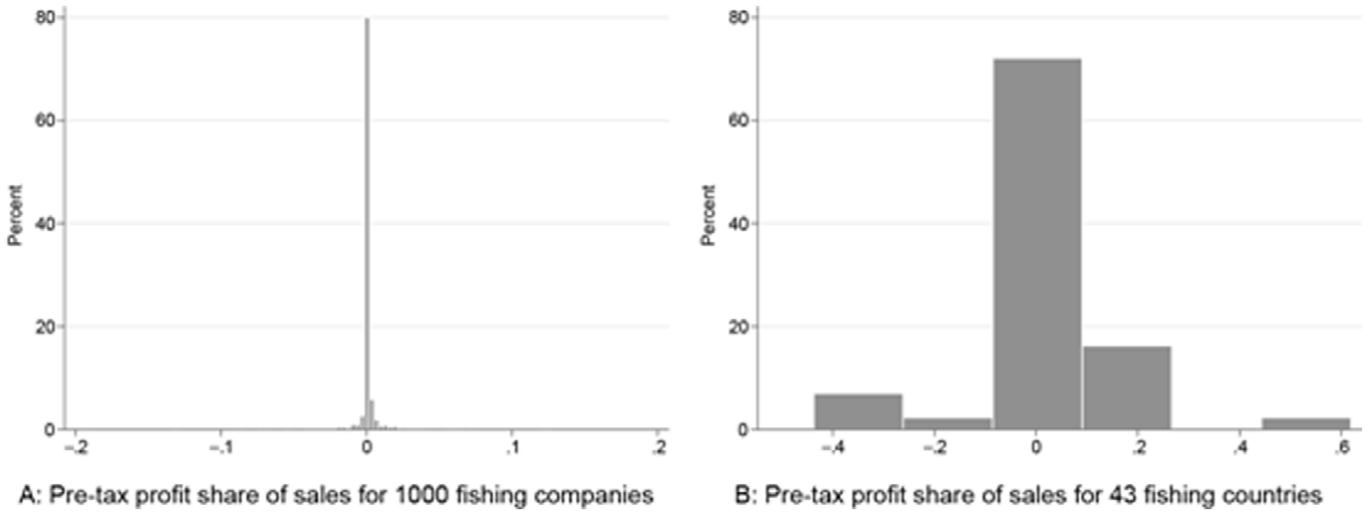
Histograms of pre-tax profit share of total sales for a sample of 1000 global fishing companies. Figure taken from [24].

A recent fishing industry report [24] provides financial data over 3 years (up to 2009), including pre-tax profit for the top 1000 commercial fishing companies worldwide. The numbers in this report support some of the counterintuitive outcomes of our study. These 1000 companies operate in 43 countries on all continents. The total annual sales value for all companies is about US$21 billion or 25% of estimated landed value worldwide. Of these 1000 companies, 339 reported negative annual pre-tax profits. Thirty-one of the 43 countries have at least one company reporting negative pre-tax profits, and of the 12 countries that report only positive pre-tax profits, nine countries had only one company in the dataset, suggesting that the optimistic results for these countries may be a result of limited data. Sixteen of the 43 countries for which data were reported had negative average pre-tax profits at the aggregate national level, at which the average ratio of pre-tax profit to sales volume is only marginally greater than zero (Figure 4). These data present an interesting and more micro-level view of the industry that is complementary to our estimates, showing that within the same country, some firms may be quite profitable, while others are much less so, resulting in negative aggregate profit at the national level.

## Materials and Methods

To estimate the potential gains from rebuilding global fisheries, we use estimates of catch loss [19], defined as the difference between current landings and Maximum Sustainable Yield (MSY) for those species that are considered to be over-exploited. It should be noted that MSY does not maximize economic yield (MEY) except when the stock size of fish does not affect the cost of fishing, and discount rate is zero. Still, we apply MSY in this analysis for practical and policy reasons, as it is a stipulated target or management reference point for many national legislations and international conventions. Other assumptions made in our analysis are: (i) the real ex-vessel fish price is constant through time (they have remained relatively stable since 1970) [16], [25]; (ii) during rebuilding, the costs of fishing change in proportion to changes in effort; (iii) the costs of fisheries management increase by 25% to US$10 billion a year, to support effective management under a rebuilt scenario; and (iv) the reported US$19 billion of annual harmful and ambiguous subsidies [17] are eliminated, since providing capacity-enhancing subsidies is fundamentally at odds with rebuilding fisheries. We also assume a rebuilding period of 10 years (e.g., Magnuson–Stevens Fishery Conservation and Management Act of the USA). Further support for this assumption is given by Costello et al. [26], who found that under an optimal rebuilding strategy, stock recovery requires between 4 and 26 years (with a mean of 11 years), depending on the fish species.

### 1. Estimating Global Fleet Size

The FAO estimates that there are currently 35 million people engaged in capture fisheries on either a part- or full-time basis [3]. The same report indicates that 90% of these fishers participate in the small-scale sector, while the remaining 10% can be classified as large-scale. The FAO also reports that the world’s fishing fleet is comprised of 4.3 million vessels, 59% of which are motorized and 14% of motorized vessels (8% of all vessels) are greater than 12 meters in length [3]. In this study, we take a broad definition of large-scale vessels that includes all motorized vessels over 12 m in length, which is of sufficient size to represent considerable fishing pressure and potential impact on the environment. Under this criterion, we estimate the number of large-scale fishing vessels worldwide to be 355,000, with the remaining 3.94 million vessels are classified as small-scale.

### 2. Estimating Effort Reductions Required to Rebuild Global Fisheries

**Figure 5:**
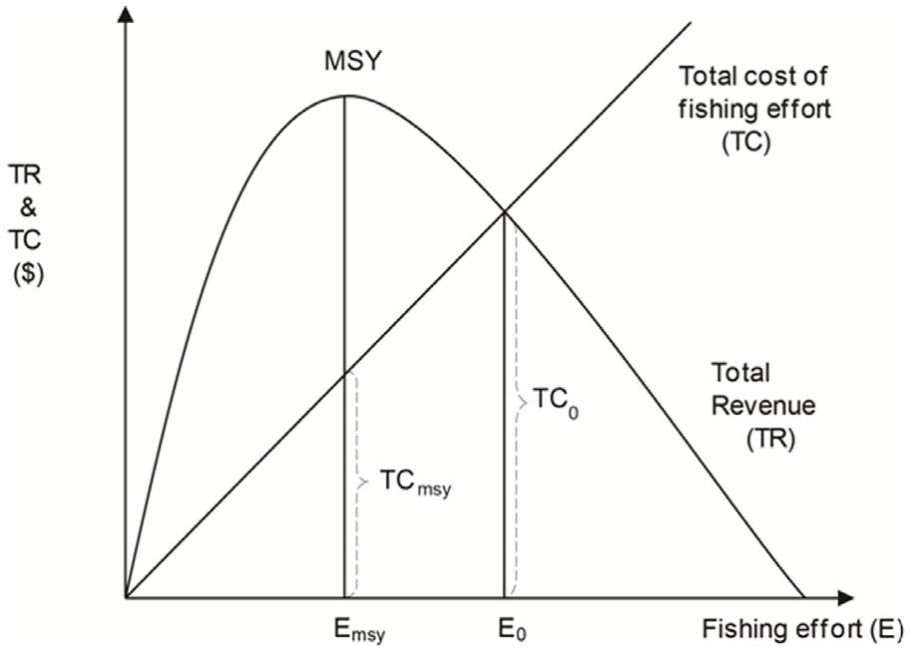
The Schaeffer surplus-production model, based on Gordon [**27**].

We model the global fishery using the Schaefer surplus-production model commonly applied to single-stock fisheries [27]. Since many fish stocks around the globe are either fully- or over-exploited, the global fishery is currently using more effort than needed to produce maximum sustainable yields (*E_MSY_* in Figure 5), which we use as a global proxy for sustainable fisheries. We are cognizant of the diversity of fisheries management uses of biomass levels with MSY (either slightly above or below) as management reference or target points. In order to achieve maximum sustainable yields, effort will need to be reduced from current levels (e.g., *E_0_* in Figure 5) to a lower level that is consistent with maximum sustainable yield (*E_msy_*). At *E_msy_*, the total cost of fishing is reduced from *TC_0_* to *TC_msy_*. For our calculations, we make the simplifying assumption that there is no substitution between labor and capital, so the shares of components of fishing costs (i.e., fuel, wages, etc.) remain constant.

Recognizing that large- and small-scale fisheries have different fishing power, and in order to minimize the effect of effort reductions on fishers (labor), who are predominantly in the small-scale sector, we weigh effort reductions more heavily on large-scale operations. We express total fishing effort in the global fishery as:
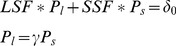
(1)where *LSF* and *SSF* are the number of large- and small-scale fishers, respectively. The parameters *P_l_* and *P_s_* represent the fishing power of large- and small-scale fishers, while *γ* represents the power of large-scale fishers relative to small-scale fishers. Total current fishing effort is *δ_0_*. By re-expressing *LSF*, *SSF* and *δ_0_* as terms that are relative to the total current fishing effort (i.e., dividing both LHS and RHS of eq. 1 by *δ_0_*), we have:



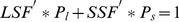
(2)Pauly [28] reports an estimate of *γ,* which places the fish catching power of large-scale fishers at 18 times that of their small-scale counterparts. This leaves us with a system of two equations with two unknowns that can be solved for *P_l_* and *P_s_*, which are used to estimate the proportions of large- and small-scale fishers required to reduce overall fishing effort:
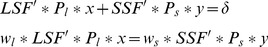
(3)


The parameters *LSF’*, *SSF’*, *P_l_* and *P_s_* are defined as in the system of equations (1 & 2) above, while *δ* represents the ratio of current effort required to rebuild fisheries, while *w_l_* and *w_s_* represent the weight of effort cuts levied on large- and small-scale fishers, respectively. The parameters *x* and *y*, which represent the proportion of large- and small-scale fishing activity to be cut, are estimated from equations (3) and used to estimate the total reductions in large- and small-scale fishers as:
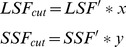
(4)


**Figure 6:**
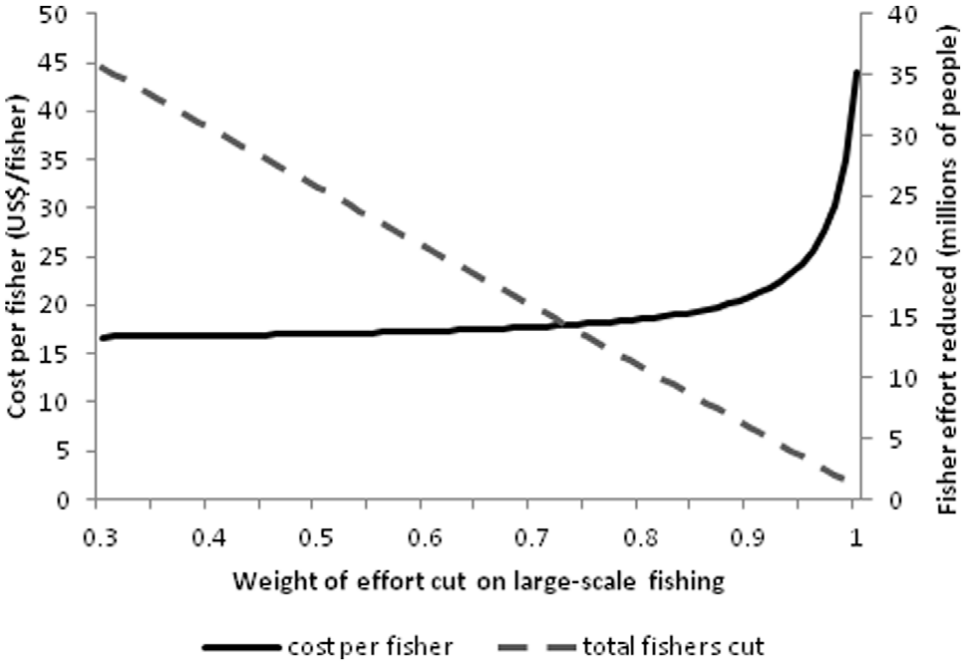
Trade-offs between reductions in cost of fishing effort and total fishing effort (in terms of number of fishers) reduced as the weight of effort cuts on large-scale fishing varies.

Lastly, we use our earlier estimates of the current number of large- and small-scale fishers and fishing vessels to estimate the number of large- and small-scale fishers and vessels that must be removed from the global fishery corresponding to our estimates of required reductions. We explore a range of weights (*w_l_* and *w_s_*) that represent equivalent total effort reductions. As can be seen in Figure 6, the trade-off between the cost of fishing effort reduction per fisher is non-linear, while the number of total fishers reduced is linear in the weighting placed on large-scale fishing effort. We suggest that by placing 80% of the weight of fishing effort reduction on large-scale fishing operations, it is consistent with cutting 60% of large-scale and 30% of small-scale fishing activity.

### 3. Estimating the Potential Value of Rebuilt Fisheries

**Figure 7:**
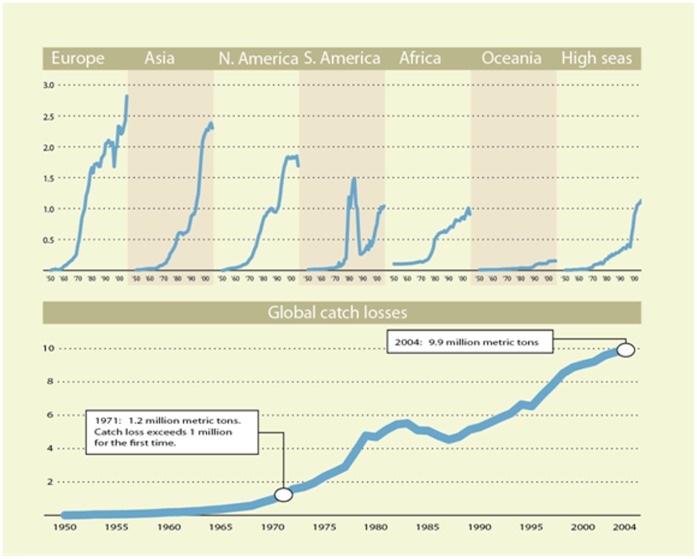
Lost catch potential due to overfishing for the six FAO regions of the world (top) and worldwide (bottom). Figure drawn using results reported in Srinivasan et al. [19].

For our present purposes, we assume that the estimated catch losses to overfishing reported by Srinivasan et al. [19] (Figure 7) may be fully regained after a period of rebuilding fisheries worldwide. To calculate potential catch losses, Srinivasan et al. [19] used catch time series from the *Sea Around Us* project for 1,066 taxa of fish and invertebrates in 301 EEZs, along with an empirical relationship they derived from catch data and stock assessments for 26 Northeast U.S. species from the U.S. National Oceanic and Atmospheric Administration (NOAA). The log-linear relationship that they found between a species’ mean maximum catch *C_max_* from catch data and its maximum sustainable yield (MSY) from stock assessment was robust (R^2^ = 0.84, p<0.001), and has since been tested for 50 fully assessed stocks in the Northeast Atlantic, where variation in MSY accounted for 98% of the variability in *C_max_* [29]. Therefore, given the dearth of detailed stock assessments for the majority of species in the world’s fisheries, Srinivasan et al. [19] applied the relationship they derived (with a 50% prediction interval) to estimate MSY levels for all stocks they identified as overfished. By comparing with reported catch levels, they arrived at estimates of lost catch by mass, reporting that without overfishing, potential landings worldwide in the year 2000 may have been 9.1 million t higher than current landings (50% prediction interval: 3.6 to 19 million t higher).

To calculate the value of these potential landings under rebuilt global fisheries, Srinivasan et al. [19] used a database of ex-vessel fish prices by Sumaila et al. [16]. For each taxon-EEZ pair designated as overfished, a price-per-tonne *p* for the maximum sustainable yield (MSY) was set by taking a weighted average of the actual prices corresponding to catches of the taxon within ±30, ±50, or ±100% of the estimated MSY level, in order of preference depending on data availability. This approach was used to account for the impacts of overfishing, and thus scarcity, on price levels.

There is debate among fisheries scientists as to the reliability of overfishing estimates based on catch trends rather than stock assessments, with some arguing that catch-based approaches are prone to overestimate depletion [30]. Srinivasan et al. [19] were careful to avoid the biases described by Branch et al. [30], with the result that the former’s estimate of the percentage of overfished stocks worldwide (16–31%) was similar to, but more conservative than, that reported by Branch et al. (28–33%), and similar also to a recent assessment by the FAO [31]. Indeed, Froese et al. [29] demonstrated that both stock- and catch-based assessments of overfishing in the Northeast Atlantic show the same trends, although the catch-based methods were generally late to recognize declines in biomass. Thus, a catch-based method would underestimate lost catch, i.e., the direct opposite direction of the bias over which Branch et al. [30] have expressed concern. Moreover, Worm et al. [6] compared areas where there were both detailed stock assessment information and more general data including catch time series, and found that catches follow biomass trends, if belatedly.

**Table 2:** Wages, normal profit and resource rent for current fisheries by FAO region.

Region	Wages	Earnings/Normal Profit*	Resource rent
	(US$ billions)		
Africa	1.51	0.60	−2.63
Asia	14.93	3.13	−4.73
Europe	6.77	0.77	−4.32
North America	4.72	1.12	1.13
Oceania	1.38	0.16	−0.58
South America	1.65	0.30	−1.80
**World Total**	30.96	6.08	−12.93

* Profit is defined here as the return to capital or normal profit, i.e., payments to owners of capital.

Based on Costello et al. [26], who estimated the recovery time for 18 simulated fish species to be 11 years on average, with a range of 4–26 years depending on the species, we assume a rebuilding period of 10 years (*t* = 0–9) in this study. During this period, we assume that the only gains to occur are those from a reduction in the current net resource rent loss from negative US$13 billion per year to zero. Following modelling work reported in UNEP’s Green Economy Report [32], we also assume that global fisheries landings decline linearly from ∼80 to 50 million tonnes per year from *t* = 0–5 as fishing effort declines, but then rise linearly to the rebuilt level (∼90 million tonnes) by *t* = 9. Once global fisheries have been rebuilt, this potential gain in resource rent would recur annually into perpetuity; here we consider only the flow for the subsequent 40 years after rebuilding (*t* = 10–49).

We estimate *R,* resource rent adjusted for subsidies, as follows:
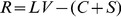
(5)where *LV* represents the landed value of officially reported marine landings. The total variable cost of fishing is represented by *C* and subsidies are represented by *S*.

The computed resource rents for the six major Food and Agriculture Organization of the United Nations (FAO) regions (Africa; Asia; Europe; North America; Oceania; South and Central America plus the Caribbean) are summarized in Table 2.

We compute the gains from rebuilding (*P_gains_*) as the value of the rebuilt resource rent (*R_rebuilt_*) minus the value of current resource rent (*R_current_*):
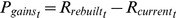
(6)where *t* represents time. We assume that globally, rebuilt fisheries will be successful in avoiding subsequent unsustainable increases in effort.

We calculate the present value of net gains from rebuilding global fisheries as follows:
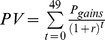
(7)where *PV* is the present value of the net gain in resource rent, *r* is the prevailing rate of discount and *t* represents time from present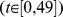. In our analysis, we assume a fixed discount rate of *r* = 0.03 (i.e., 3%) and compute the present value of net gains in resource rent for 50 years after rebuilding. We use this discount rate because many environmental economists have argued for and applied lower-than-market rates due to the central role of environmental resources in ensuring sustainable economies through time [33]–[35] Changes in the value of fisheries landings, costs, subsidies and resource rent through the transition time period are summarized in Figure 3.

**Table 3:** Wages, normal profit, resource rent and increase in rent from rebuilt fisheries.

Region	Wages	Earnings/Normal Profit*	Resource rent	Increase in rent
	(US$ billions)			
Africa	0.76	0.30	0.85	3.48
Asia	7.46	1.56	30.82	35.54
Europe	3.38	0.39	8.80	13.12
North America	2.36	0.56	7.98	6.86
Oceania	0.69	0.08	2.73	3.31
South America	0.83	0.15	2.51	4.31
**World Total**	15.48	3.04	53.69	66.61

* Profit is defined here as the return to capital or normal profit, i.e., payments to owners of capital.

### 4. Estimating the Cost of Rebuilding Global Fisheries

In addition to differences between current resource rent and that which is captured during the period of rebuilding, we estimate the costs necessary to reduce fishing capacity to levels required to allow fish stocks to rebuild. These costs are estimated based on the cost of effort reductions described earlier in the methods. We estimate wages, profits, resource rent and increase in resource rent from rebuilding for the six major FAO regions (Africa; Asia; Europe; North America; Oceania; South and Central America plus the Caribbean) in Table 3.

Since the real cost of rebuilding fisheries is foregone resource rent that may occur as fishing effort is reduced initially, we estimate the cost of rebuilding global fisheries through the transition to rebuilt fisheries as the difference between current fisheries resource rent and that which is realized through the period of transition. We hold the assumption that all harmful capacity-enhancing and ambiguous subsidies (Table 4) must be cut immediately or re-directed to make them beneficial subsidies, e.g., by investing in managing the rebuilding process.

**Table 4:** Annual global fisheries subsidies by category [17].

Category	Subsidies (US$ billions)
Beneficial^a^	8
Harmful^b^	16
Ambiguous^c^	3
**Total**	27

a Lead to ‘investment’ in the natural capital of fishery resources. They enhance the growth of fish stocks through conservation programs, and control and surveillance measures.

b Lead to ‘disinvestments’ in the natural capital of the fishery resources, including all forms of capital inputs and infrastructure investments from public sources that reduce cost or enhance revenue.

c Have the potential to lead to either ‘investment’ or ‘disinvestment’ in the fishery resources, and lead to resource enhancement or to resource overexploitation.

### 5. Calculating the Unit Cost of Reducing Fishing Effort

Policy makers generally prefer to minimize the employment impact of rebuilding fisheries. It would therefore be attractive to target effort reductions on large-scale vessels only, as they employ less people per unit of fish landed [36]. While the goal of matching global fishing capacity with the productive capacity of the resource by cutting only large-scale vessels seems theoretically possible, it would be ineffective in areas that are overfished but dominated by small-scale vessels. Available gear-related data [36], [37] reveal that the split between large- and small-scale vessels in the developed world is about 50∶50, while it is 25∶75 in developing countries. Our analysis of fishing effort cuts show that permanently removing around 213,000 large-scale and 1.2 million small-scale vessels (60% and 30% reductions, respectively), would halve the world’s fishing capacity. This weighting between large- and small-scale fishing capacity is supported by evidence that large-scale vessels currently land roughly two-thirds of the world’s annual reported marine landings [28].

**Figure 8:**
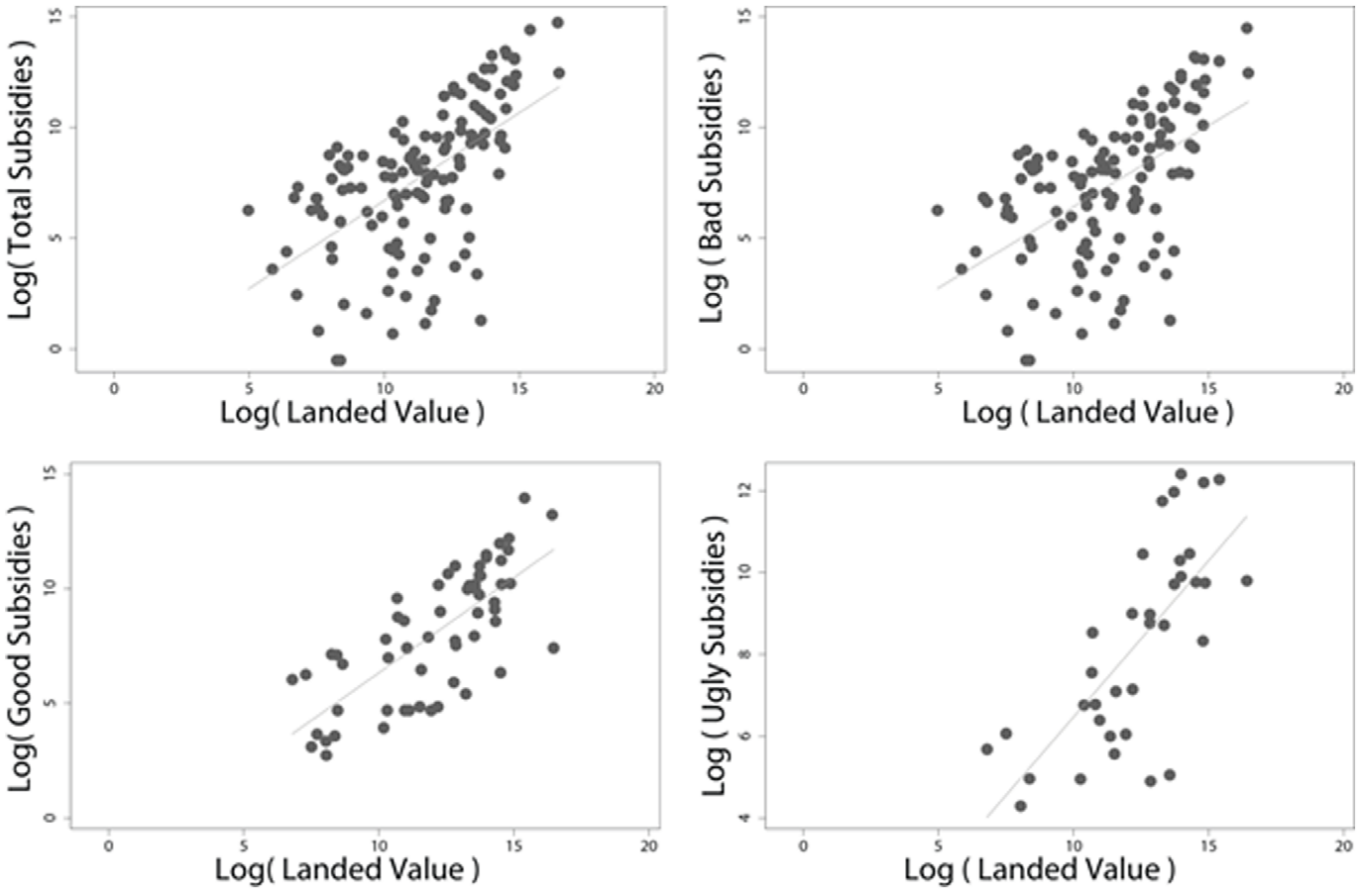
Correlation between reported subsidies [**17**] and landed-value [**16**].

Cost data [18] reveal that crew from large- and small-scale fisheries earn, on average, wages of US$20,000 and US$10,000 per year, respectively. Furthermore, vessels in large- and small-scale fisheries pay, on average, US$11,000 and US$2,500 per year for capital. Based on vessel and crew data from the European Union [38], we estimate that the average cost of a vessel buyback is roughly equal to the average interest payments on a vessel for five years, and the average cost of crew retraining is estimated at 1.5 times the average annual crew wages. Therefore, the average cost of decommissioning large- and small-scale fishing vessels would be US$55,000 and US$12,500, respectively. Likewise, payout/retraining costs for large- and small-scale fishers to leave fishing permanently would be US$30,000 and US$15,000 per person, respectively. Clearly, decommissioning costs for the extremely large industrial vessels with global roaming abilities would be higher than the above vessel averages.

**Table 5:** Global cost (mean +/−95% CI) of fishing (Year 2005 US$ per t of catch), separated into variable and fixed cost component [18].

	Lower 95% CI	Mean	Upper 95% CI
Cost category	(US$ per t)		
Variable	639	928	1,413
Fixed	123	192	164
**Total**	**762**	**1,120**	**1,477**

### 6. Data and Databases

We utilize four interrelated global databases of fisheries statistics, namely, databases of fisheries landings, ex-vessel fish prices, subsidies, and fishing costs. Each database represents the work of an international team of fisheries scientists and economists, and collectively represents the world’s most comprehensive collection of truly global fisheries (economic) data.

Our catch data, the main source of which is the FAO global capture production database supplemented by several more detailed regional catch data sources, allocates the reported fish landings to a global system of 30-minute latitude by 30-minute longitude cells (just under 180,000 marine cells globally) using the intersection of statistical reporting areas, biological taxon distributions of reported taxa, general habitat preferences, global fishing access agreements and fishing patterns of reporting countries. Details of the methods and procedures of this spatial allocation are described in Watson et al. [15], and country-specific data by FAO region are presented in Tables S1, S2, S3, S4, S5 and S6.

The global ex-vessel fish price database used here, described by Sumaila et al. [16], covers annual average ex-vessel prices for all marine fish taxa by country reported as caught from 1950 to 2006. Through their extensive search of publicly available, but widely scattered and incompatible, national and regional statistical reports and grey literature, Sumaila et al. [16] accumulated over 31,000 records of observed ex-vessel prices in 35 countries, representing about 20 percent of the global landings over the 60 year period. In order to ‘fill the gaps’ in the database, a series of rules were developed whereby all catches with no reported prices were inferred to have an estimated price computed from the reported prices from related taxa, similar markets or years. Since the database was first presented, new reported prices have been included from various additional sources, and rules as to how prices relate across taxa, markets or years have been modified to improve the quality of the estimated prices. The time series of landed values of the world’s marine fisheries, computed through the combination of the spatially allocated catch data with the ex-vessel price database (country-specific landed values by FAO region) are presented in Tables S1, S2, S3, S4, S5 and S6, and have been used in various analyses, such as the estimation of global subsidies [17] and costs of marine protected areas [39].

**Table 6:** Sensitivity of present value of rebuilding costs to parameter assumptions.

Effort reduction to achieveMSY (%)	Adjustment costs (US$ billions)*		
	−20%	Mean	+20%
40	130	162	195
50	162	203	243
60	195	243	292

* Adjustment costs include the cost of vessel buybacks and payouts for fishers to ease the transition to alternate employment.

The fisheries subsidies database defines subsidies as financial transfers, directly or indirectly, from government to the fishing industry [17]. This database is the most comprehensive collection of publicly available data on fisheries subsidies at the global level, spanning the years 1990 to 2006. Each record in the database represents expenditure in one of twenty-six identified subsidy categories for a given country and year combination. Where qualitative information indicates the presence of a subsidy program, yet quantitative data are not available, the database records the expenditure data as ‘missing’ for later estimation.

**Table 7:** Sensitivity of present value potential gain in resource rent from rebuilding to parameter assumptions.

Effort reductions to achieveMSY (%)	Potential gains (US$ billions)*		
	Lower 95% CI	Mean	Upper 95% CI
40	976	1,128	1,428
50	821	972	1,272
60	665	816	1,117

* Gains are denoted as the present value of increased resource rent from rebuilt fisheries.

Estimation of ‘missing’ subsidy data follows the method of Sumaila et al. [17] who utilize the strong relationship between fisheries subsidies and landed value (Figure 8) to estimate subsidy expenditure in cases where programs are documented without quantitative information. We use this procedure to estimate existing but unquantified fisheries subsidies for any of the twenty-six subsidy categories in any of the 144 maritime countries of the world, and summarize these globally by three general categories (Table 4): ‘beneficial’ (lead to ‘investment’ in the natural capital of fishery resources, thus enhancing growth of fish stocks through conservation programs, and control and surveillance measures), ‘harmful’ (lead to ‘disinvestments’ in the natural capital of the fishery resources, including all forms of capital inputs and infrastructure investments from public sources that reduce cost or enhance revenue) and ‘ambiguous’ (have the potential to lead to either ‘investment’ or ‘disinvestment’ in the fishery resources, and lead to resource enhancement or to resource overexploitation). Fuel- and non-fuel subsidies by country within FAO regions are summarized in Tables S1, S2, S3, S4, S5 and S6.

Lam et al. [18] developed a global database of fishing costs by country and gear types, capturing two types of fishing cost, variable (operating) and fixed costs in 144 maritime countries, representing approximately 98% of global landings in 2005. Results from this database are summarized in Table 5. Each record in the database represents a country and gear type combination. The gear types included in the database are based on the gear categorization system of the *Sea Around Us* project [40].

Fishing cost data were collected from secondary sources in major fishing countries in each of the six FAO regions. In order to include as many data of observed cost as possible, Lam et al. [18] accessed all available sources, irrespective of publication year, thus extending their efforts in collecting cost data from 1950 to the most recent year for which data were available. The data were then converted to 2005 real values using the consumer price index (CPI) for each country obtained from the World Bank. To make the comparison of fishing cost among different regions and countries possible, they converted all fishing costs from local currencies to US dollars by using currency exchange rates provided by the World Bank, and standardized the original cost to annual cost in US$ per tonne of catch.

A process of progressive refinement [16], [18] was then used to estimate the cost of all gear types in each fishing country from the observed, collected cost. Therefore, Lam et al. [18] ensured that all gear types in each maritime country of the world were assigned a cost, either the observed value where available, or an appropriate average estimated cost. Variable fishing costs by country within FAO region are summarized in Tables S1, S2, S3, S4, S5 and S6.

### 7. Sensitivity Analysis

We test the sensitivity of our results both in terms of the benefits and costs of rebuilding. An estimate of the potential contribution of rebuilt fisheries from Srinivasan et al. [19] is 89 million t per year (50% prediction interval: 83 million to 99 million t per year) and US$100 billion per year in landed value (50% prediction interval: US$93 billion to US$116 billion per year). This represents an increase over the current value of fisheries landings of US$13 billion per year (range: US$5 billion to US$29 billion per year). We also test the sensitivity of our estimates to changes in the cost of fishing by allowing the needed effort reductions to attain maximum sustainable yield to be within a range of 40–60% of current fishing effort, with a mean value of 50% of current effort levels.

Table 6 presents the results of the sensitivity analysis with respect to the costs of adjusting factors of fisheries production to maximum sustainable yield. The top-left and bottom-right cells of this table display the best- and worst-case scenarios for our estimates of adjustments costs. Table 7 presents the results of our sensitivity analysis on the estimates for rebuilding benefits in a three-by-three matrix, thus showing potential best- and worst-case scenarios of costs of rebuilding fisheries in the bottom-right and top-left cells of the table, respectively. It can readily be seen from these two tables that, even under drastic changes in our estimates, the combination of the worst-case scenarios result in large net present value of resource rent from rebuilt global fisheries.

## Supporting Information

Table S1 **Key fisheries data (annual averages for 2000s) for Africa.**
(DOCX)

Table S2 **Key fisheries data (annual averages for 2000s) for Asia.**
(DOCX)

Table S3 **Key fisheries data (annual averages for 2000s) for Europe.**
(DOCX)

Table S4 **Key fisheries data (annual averages for 2000s) for North America.**
(DOCX)

Table S5 **Key fisheries data (annual averages for 2000s) for Oceania.**
(DOCX)

Table S6 **Key fisheries data (annual averages for 2000s) for South, Central America and the Caribbean.**
(DOCX)
