# Supplementary material for: Benefits of Rebuilding Global Marine Fisheries Outweigh Costs
Source: PLoS One. 2012 Jul 13;7(7):e40542. doi: 10.1371/journal.pone.0040542 (PMC3396648; doi:10.1371/journal.pone.0040542)
Supplement: Table S1 — Key fisheries data (annual averages for 2000s) for Africa. (DOCX) [file pone.0040542.s001.docx]

| **Country** | **Landings (t x 10^3^)** | **Landed-value** | **Variable Cost** | **Subsidies** |
| --- | --- | --- | --- | --- |
|  |  | **(US$ million)** | | |
| Algeria | 126.26 | 26.11 | 115.94 | 6.69 |
| Angola | 217.00 | 122.98 | 233.03 | 74.53 |
| Eritrea | 4.03 | 2.62 | 4.12 | 2.00 |
| Cameroon | 67.33 | 64.53 | 74.50 | 9.45 |
| Cape Verde | 8.79 | 4.69 | 11.86 | 11.22 |
| Comoros | 15.07 | 4.68 | 22.63 | 0.68 |
| Congo Rep | 25.80 | 13.05 | 27.23 | 1.85 |
| Congo Dem Rep | 5.00 | 1.83 | 5.00 | 0.17 |
| Benin | 9.81 | 4.06 | 11.21 | 6.59 |
| Eq Guinea | 1.90 | 0.43 | 1.95 | 0.25 |
| Djibouti | 0.26 | 0.15 | 0.41 | 0.56 |
| Gabon | 33.47 | 26.75 | 38.08 | 12.62 |
| Gambia | 30.45 | 26.08 | 32.14 | 12.14 |
| Ghana | 316.80 | 105.33 | 359.72 | 32.85 |
| Guinea | 92.57 | 83.51 | 91.55 | 28.91 |
| Cote d'Ivoire | 26.88 | 13.92 | 26.53 | 12.28 |
| Kenya | 7.11 | 5.50 | 9.54 | 4.83 |
| Liberia | 8.77 | 4.83 | 10.72 | 0.61 |
| Libya | 46.07 | 25.57 | 45.21 | 5.09 |
| Madagascar | 104.86 | 102.47 | 120.67 | 12.86 |
| Maldives | 185.98 | 638.19 | 257.68 | 65.17 |
| Mauritania | 291.88 | 354.37 | 273.84 | 25.99 |
| Mauritius | 9.85 | 5.52 | 14.49 | 2.23 |
| Morocco | 1,023.34 | 350.65 | 942.24 | 91.74 |
| Mozambique | 29.51 | 31.55 | 39.13 | 21.54 |
| Namibia | 551.18 | 322.08 | 525.24 | 122.46 |
| Nigeria | 284.18 | 189.47 | 315.42 | 30.95 |
| GuineaBissau | 5.93 | 4.92 | 5.97 | 4.39 |
| Sao Tome Principe | 3.84 | 1.83 | 4.52 | 0.74 |
| Senegal | 358.18 | 111.19 | 176.40 | 70.53 |
| Seychelles | 106.93 | 28.40 | 178.15 | 28.65 |
| Sierra Leone | 131.78 | 97.08 | 132.52 | 13.66 |
| Somalia | 29.80 | 33.95 | 29.07 | 4.28 |
| South Africa | 816.26 | 400.05 | 694.87 | 69.61 |
| Sudan | 5.50 | 5.61 | 5.32 | 1.28 |
| Togo | 22.73 | 4.10 | 19.71 | 1.54 |
| Tunisia | 107.71 | 52.50 | 114.22 | 26.48 |
| Egypt | 107.45 | 72.35 | 118.13 | 15.84 |
| Tanzania | 54.97 | 26.45 | 62.23 | 9.97 |
| **Total** | **5,275.23** | **3,369.36** | **5,151.17** | **843.24** |
