# Supplementary material for: Benefits of Rebuilding Global Marine Fisheries Outweigh Costs
Source: PLoS One. 2012 Jul 13;7(7):e40542. doi: 10.1371/journal.pone.0040542 (PMC3396648; doi:10.1371/journal.pone.0040542)
Supplement: Table S2 — Key fisheries data (annual averages for 2000s) for Asia. (DOCX) [file pone.0040542.s002.docx]

| **Country** | **Landings (t x 10^3^)** | **Landed-value** | **Variable Cost** | **Subsidies** |
| --- | --- | --- | --- | --- |
|  |  | **(US$ million)** | | |
| Bahrain | 11.50 | 27.04 | 11.12 | 11.92 |
| Bangladesh | 474.60 | 239.17 | 226.28 | 62.83 |
| Brunei Darsm | 2.39 | 1.66 | 1.79 | 0.77 |
| Cambodia | 60.00 | 100.94 | 50.07 | 7.37 |
| China Main | 9,945.33 | 14,056.08 | 9,306.69 | 4,139.47 |
| India | 3,177.24 | 2,765.22 | 2,258.53 | 1,070.17 |
| Indonesia | 4,389.76 | 2,446.88 | 4,294.60 | 989.70 |
| Iran | 342.30 | 830.92 | 338.35 | 243.09 |
| Israel | 4.56 | 5.45 | 2.09 | 1.22 |
| Japan | 4,028.59 | 10,181.01 | 5,801.93 | 4,636.02 |
| Jordan | 0.16 | 0.26 | 0.16 | 0.07 |
| Korea Rep | 1,630.65 | 2,312.40 | 3,251.19 | 893.94 |
| Kuwait | 4.90 | 8.93 | 5.70 | 1.00 |
| Lebanon | 3.52 | 4.57 | 2.74 | 0.59 |
| Malaysia | 1,203.27 | 1,348.37 | 1,143.01 | 317.23 |
| Myanmar | 1,226.73 | 780.70 | 492.24 | 157.76 |
| Oman | 150.57 | 232.67 | 144.97 | 79.50 |
| Pakistan | 339.83 | 470.04 | 280.92 | 136.71 |
| Philippines | 2,103.25 | 2,066.25 | 1,941.51 | 918.83 |
| Qatar | 13.94 | 16.14 | 16.71 | 3.76 |
| Saudi Arabia | 54.36 | 98.23 | 61.15 | 33.31 |
| Singapore | 1.92 | 2.88 | 1.80 | 0.32 |
| Sri Lanka | 136.12 | 353.59 | 154.79 | 132.35 |
| Suriname | 28.04 | 80.60 | 23.54 | 15.83 |
| Syria | 3.68 | 4.67 | 3.37 | 0.78 |
| Taiwan | 1,016.41 | 2,570.88 | 2,227.11 | 360.48 |
| Thailand | 2,578.86 | 1,953.37 | 1,236.87 | 552.60 |
| Turkey | 379.84 | 405.66 | 232.42 | 97.06 |
| United Arab Em | 86.67 | 95.26 | 86.98 | 10.59 |
| Viet Nam | 1,791.10 | 1,869.52 | 1,024.47 | 697.41 |
| Yemen | 263.00 | 540.47 | 282.60 | 117.56 |
| **Total** | **35,453.07** | **45,869.83** | **34,905.69** | **15,690.25** |
