# Supplementary material for: Benefits of Rebuilding Global Marine Fisheries Outweigh Costs
Source: PLoS One. 2012 Jul 13;7(7):e40542. doi: 10.1371/journal.pone.0040542 (PMC3396648; doi:10.1371/journal.pone.0040542)
Supplement: Table S3 — Key fisheries data (annual averages for 2000s) for Europe. (DOCX) [file pone.0040542.s003.docx]

| **Country** | **Landings (t x 10^3^)** | **Landed-value** | **Variable Cost** | **Subsidies** |
| --- | --- | --- | --- | --- |
|  |  | **(US$ million)** | | |
| Albania | 2.06 | 2.55 | 2.92 | 1.34 |
| Belgium | 24.10 | 103.88 | 72.07 | 17.07 |
| Bulgaria | 3.41 | 2.58 | 3.59 | 0.79 |
| Croatia | 34.64 | 19.47 | 41.61 | 1.80 |
| Cyprus | 1.78 | 3.63 | 2.76 | 1.44 |
| Denmark | 1,722.58 | 1,970.60 | 898.14 | 168.01 |
| Estonia | 95.32 | 62.11 | 97.19 | 17.41 |
| Finland | 87.69 | 21.63 | 113.55 | 30.58 |
| France | 620.56 | 1,423.08 | 837.45 | 436.36 |
| Georgia | 2.94 | 1.21 | 3.46 | 0.97 |
| Germany | 263.18 | 247.40 | 328.99 | 72.09 |
| Greece | 144.17 | 422.21 | 280.67 | 148.35 |
| Iceland | 1,660.55 | 876.80 | 2,213.24 | 144.76 |
| Ireland | 262.01 | 245.61 | 481.02 | 101.50 |
| Italy | 294.74 | 1,337.05 | 703.23 | 240.42 |
| Latvia | 150.06 | 52.53 | 163.81 | 20.25 |
| Lithuania | 138.13 | 81.47 | 157.90 | 18.32 |
| Malta | 1.44 | 1.95 | 2.56 | 1.59 |
| Netherlands | 550.34 | 634.00 | 654.10 | 24.80 |
| Norway | 2,390.90 | 2,109.10 | 1,604.15 | 286.35 |
| Poland | 132.74 | 57.63 | 142.89 | 36.76 |
| Portugal | 211.56 | 304.99 | 325.36 | 104.15 |
| Romania | 2.02 | 1.04 | 2.28 | 0.59 |
| Russian Fed | 2,979.24 | 2,803.16 | 3,438.77 | 1,481.76 |
| Spain | 851.43 | 2,106.96 | 1,603.91 | 673.66 |
| Sweden | 254.87 | 123.65 | 207.86 | 148.93 |
| UK | 768.40 | 727.90 | 874.38 | 345.68 |
| Ukraine | 238.51 | 106.33 | 339.54 | 49.72 |
| **Total** | **13,889.34** | **15,850.52** | **15,597.39** | **4,575.42** |
