# Supplementary material for: Benefits of Rebuilding Global Marine Fisheries Outweigh Costs
Source: PLoS One. 2012 Jul 13;7(7):e40542. doi: 10.1371/journal.pone.0040542 (PMC3396648; doi:10.1371/journal.pone.0040542)
Supplement: Table S4 — Key fisheries data (annual averages for 2000s) for North America. (DOCX) [file pone.0040542.s004.docx]

| **Country** | **Landings (t x 10^3^)** | **Landed-value** | **Variable Cost** | **Subsidies** |
| --- | --- | --- | --- | --- |
|  |  | **(US$ million)** | | |
| Canada | 1,087.43 | 2,964.73 | 1,679.12 | 842.30 |
| Mexico | 1,204.94 | 1,287.93 | 1,176.68 | 268.55 |
| USA | 4,792.78 | 8,029.51 | 5,377.71 | 1,812.74 |
| **Total** | **7,085.16** | **12,282.16** | **8,233.51** | **2,923.59** |
