# Supplementary material for: Benefits of Rebuilding Global Marine Fisheries Outweigh Costs
Source: PLoS One. 2012 Jul 13;7(7):e40542. doi: 10.1371/journal.pone.0040542 (PMC3396648; doi:10.1371/journal.pone.0040542)
Supplement: Table S5 — Key fisheries data (annual averages for 2000s) for Oceania. (DOCX) [file pone.0040542.s005.docx]

| **Country** | **Landings (t x 10^3^)** | **Landed-value** | **Variable Cost** | **Subsidies** |
| --- | --- | --- | --- | --- |
|  |  | **(US$ million)** | | |
| Australia | 228.83 | 853.79 | 536.68 | 481.90 |
| Fiji | 38.81 | 133.73 | 77.64 | 39.83 |
| Kiribati | 33.62 | 93.72 | 76.93 | 23.53 |
| Marshall Is | 57.16 | 494.98 | 169.94 | 72.11 |
| Micronesia | 29.33 | 245.99 | 85.69 | 170.08 |
| Nauru | 0.04 | 0.27 | 0.11 | 0.17 |
| New Zealand | 747.82 | 1,353.48 | 1,371.26 | 47.22 |
| Palau | 0.93 | 1.09 | 1.48 | 1.50 |
| Papua N Guinea | 273.66 | 449.31 | 762.47 | 662.01 |
| Samoa | 3.20 | 15.93 | 8.09 | 7.33 |
| Solomon Is. | 27.34 | 33.69 | 66.74 | 34.98 |
| Tonga | 2.00 | 8.36 | 4.65 | 7.16 |
| Vanuatu | 146.99 | 902.27 | 313.79 | 143.99 |
| **Total** | **1,589.73** | **4,586.62** | **3,475.48** | **1,691.81** |
