# Supplementary material for: Benefits of Rebuilding Global Marine Fisheries Outweigh Costs
Source: PLoS One. 2012 Jul 13;7(7):e40542. doi: 10.1371/journal.pone.0040542 (PMC3396648; doi:10.1371/journal.pone.0040542)
Supplement: Table S6 — Key fisheries data (annual averages for 2000s) for South, Central America and the Caribbean. (DOCX) [file pone.0040542.s006.docx]

| **Country** | **Landings (t x 10^3^)** | **Landed-value** | **Variable Cost** | **Subsidies** |
| --- | --- | --- | --- | --- |
|  |  | **(US$ million)** | | |
| Antigua Barbuda | 3.00 | 6.52 | 2.94 | 4.11 |
| Argentina | 895.83 | 640.49 | 497.40 | 366.83 |
| Bahamas | 11.06 | 67.87 | 7.41 | 14.27 |
| Barbados | 2.18 | 2.64 | 1.64 | 0.88 |
| Belize | 4.20 | 11.60 | 3.64 | 7.88 |
| Brazil | 506.83 | 1,142.01 | 446.38 | 413.39 |
| Chile | 4,274.13 | 1,083.15 | 1,841.87 | 93.74 |
| Colombia | 75.08 | 41.28 | 53.98 | 15.42 |
| Costa Rica | 21.34 | 41.08 | 20.32 | 17.14 |
| Cuba | 28.18 | 89.24 | 24.03 | 13.89 |
| Dominica | 0.58 | 1.16 | 0.55 | 7.26 |
| Dominican Rep. | 9.41 | 22.87 | 8.42 | 7.46 |
| Ecuador | 407.12 | 198.54 | 255.06 | 47.36 |
| El Salvador | 39.06 | 116.01 | 26.90 | 9.50 |
| Grenada | 2.05 | 3.44 | 1.71 | 5.40 |
| Guatemala | 15.25 | 32.33 | 10.17 | 8.91 |
| Guyana | 52.57 | 143.31 | 60.39 | 54.54 |
| Haiti | 8.70 | 27.47 | 9.98 | 4.40 |
| Honduras | 16.46 | 42.34 | 13.47 | 11.93 |
| Jamaica | 12.70 | 34.25 | 13.33 | 10.69 |
| Nicaragua | 29.57 | 90.60 | 21.94 | 14.75 |
| Panama | 217.36 | 265.30 | 118.57 | 50.06 |
| Peru | 9,342.11 | 838.32 | 1,933.06 | 205.49 |
| St Kitts & Nevis | 0.45 | 0.97 | 0.38 | 1.08 |
| St Lucia | 1.41 | 2.71 | 1.26 | 4.04 |
| St Vincent | 3.35 | 6.28 | 2.93 | 5.29 |
| Trinidad & Tobago | 13.41 | 23.91 | 11.98 | 11.48 |
| Uruguay | 123.78 | 115.73 | 71.24 | 11.15 |
| Venezuela | 420.91 | 283.54 | 228.78 | 64.84 |
| **Total** | **16,538.07** | **5,374.97** | **5,689.75** | **1,483.20** |
